# Experimental validation of in silico predicted RAD locus frequencies using genomic resources and short read data from a model marine mammal

**DOI:** 10.1186/s12864-019-5440-8

**Published:** 2019-01-22

**Authors:** David L. J. Vendrami, Jaume Forcada, Joseph I. Hoffman

**Affiliations:** 1 Department of Animal Behavior, University of Bielefeld, Postfach 100131, 33615 Bielefeld, Germany; 2 British Antarctic Survey, High Cross, Madingley Road, Cambridge, CB3 OET UK

**Keywords:** Restriction site associated DNA sequencing (RADseq), Restriction enzyme, Reference genome, Transcriptome assembly, PredRAD, Antarctic fur seal, Pinniped

## Abstract

**Background:**

Restriction site-associated DNA sequencing (RADseq) has revolutionized the study of wild organisms by allowing cost-effective genotyping of thousands of loci. However, for species lacking reference genomes, it can be challenging to select the restriction enzyme that offers the best balance between the number of obtained RAD loci and depth of coverage, which is crucial for a successful outcome. To address this issue, PredRAD was recently developed, which uses probabilistic models to predict restriction site frequencies from a transcriptome assembly or other sequence resource based on either GC content or mono-, di- or trinucleotide composition. This program generates predictions that are broadly consistent with estimates of the true number of restriction sites obtained through in silico digestion of available reference genome assemblies. However, in practice the actual number of loci obtained could potentially differ as incomplete enzymatic digestion or patchy sequence coverage across the genome might lead to some loci not being represented in a RAD dataset, while erroneous assembly could potentially inflate the number of loci. To investigate this, we used genome and transcriptome assemblies together with RADseq data from the Antarctic fur seal (*Arctocephalus gazella*) to compare PredRAD predictions with empirical estimates of the number of loci obtained via in silico digestion and from de novo assemblies.

**Results:**

PredRAD yielded consistently higher predicted numbers of restriction sites for the transcriptome assembly relative to the genome assembly. The trinucleotide and dinucleotide models also predicted higher frequencies than the mononucleotide or GC content models. Overall, the dinucleotide and trinucleotide models applied to the transcriptome and the genome assemblies respectively generated predictions that were closest to the number of restriction sites estimated by in silico digestion. Furthermore, the number of de novo assembled RAD loci mapping to restriction sites was similar to the expectation based on in silico digestion.

**Conclusions:**

Our study reveals generally high concordance between PredRAD predictions and empirical estimates of the number of RAD loci. This further supports the utility of PredRAD, while also suggesting that it may be feasible to sequence and assemble the majority of RAD loci present in an organism’s genome.

**Electronic supplementary material:**

The online version of this article (10.1186/s12864-019-5440-8) contains supplementary material, which is available to authorized users.

## Background

Restriction site associated DNA sequencing (RADseq) is one of a family of reduced representation sequencing approaches that have revolutionized the field of molecular ecology by allowing individuals of most organisms to be genotyped at thousands to tens of thousands of genetic markers [1, 2]. RADseq uses type II restriction enzymes to digest intact genomic DNA samples into fragments, usually shorter than 1000 bp and referred to as RAD tags, which are then massively parallel Illumina sequenced. The resulting sequence reads are then aligned either de novo or to a reference genome, to generate RAD loci that are interrogated for single nucleotide polymorphisms (SNPs). These in turn provide the basis for diverse applications from linkage and association mapping [3, 4], through elucidating population structure and patterns of introgression [5, 6] to quantifying inbreeding depression [7].

**Fig. 1:**
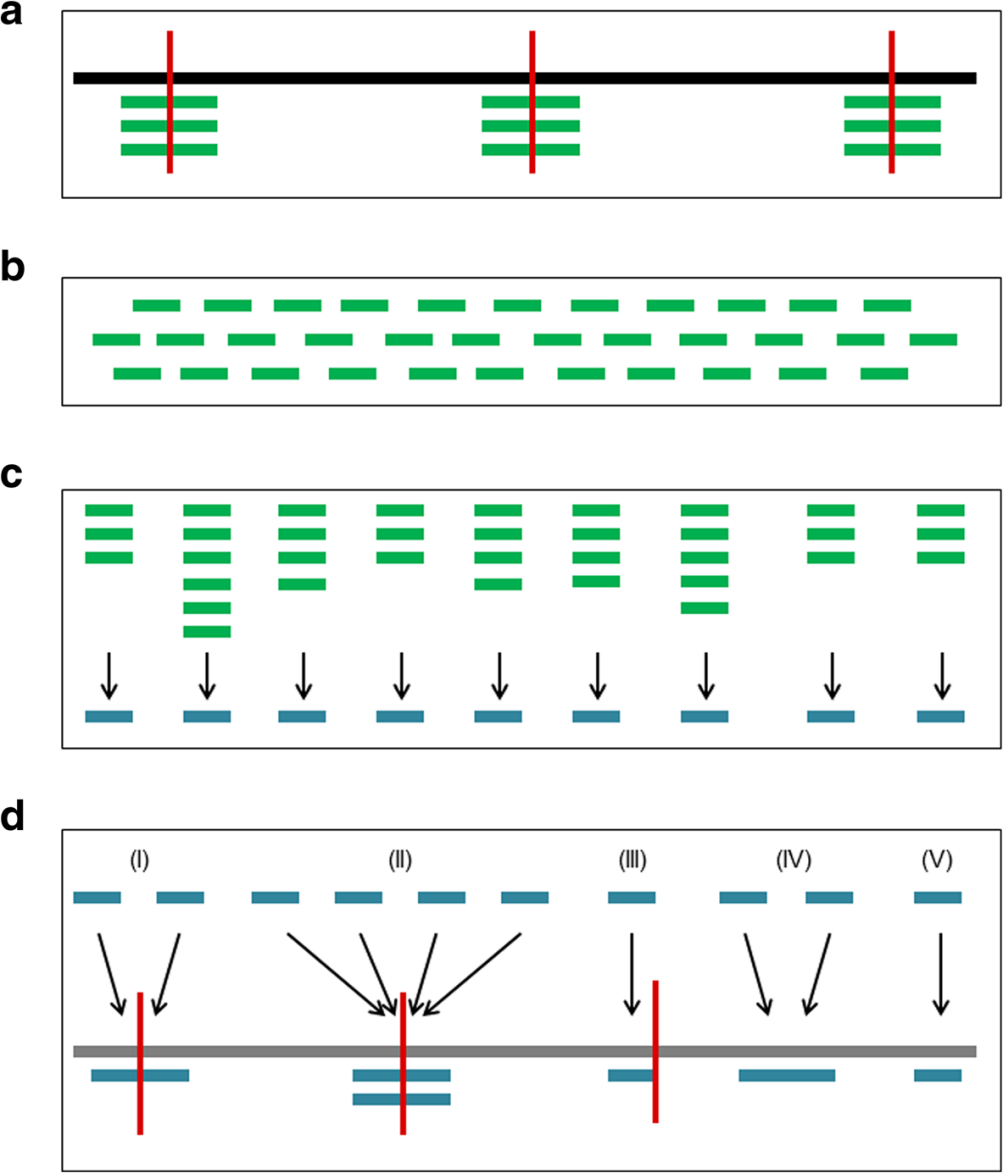
Schematic representation of the procedure used to estimate the number of cutting sites present in the Antarctic fur seal genome for the enzyme SbfI. **a** Two DNA fragments were generated for every restriction site (denoted by vertical red lines) present in the fur seal genome (denoted by a continuous black horizontal line) and the first ~ 200 bp of each of them was sequenced in multiple copies (horizontal green lines); **b** The resulting sequence data were delivered as a collection of raw sequence reads; **c** The Stacks pipeline was then employed to assemble the raw reads into RAD loci (represented by blue horizontal lines); **d** The consensus sequence of every RAD locus was mapped to the reference genome (denoted by the continuous grey horizontal line) allowing us to distinguish among five different scenarios: (I) a single pair of RAD loci map around a restriction enzyme recognition sequence; (II) multiple RAD loci map around a recognition sequence; (III) a single RAD locus maps to a recognition sequence; (IV and V) a pair or a single RAD locus maps to a genomic region not containing the recognition sequence. Scenarios I, II and III are indicative of the presence of a restriction enzyme cutting site that is represented by the assembled RADseq data

An important consideration when designing a RADseq project is the choice of which restriction enzyme to use, as this determines the number of RAD tags that will be sequenced and hence the total number of RAD loci that can be assembled. Assuming complete digestion and adequately deep and even sequencing across loci, the number of assembled RAD loci should be roughly twice the number of restriction sites, as enzymatic digestion generates two fragments that are both partially sequenced (Fig. 1). Based on this number, it is possible to calculate the amount of sequencing required to obtain a specified average depth of coverage and from there to design an optimal sequencing strategy given the available budget.

For species with genome assemblies, it is relatively straightforward to estimate the number of restriction sites for a given enzyme though in silico digestion. However, quantifying restriction site frequencies in organisms lacking genome assemblies is more problematic. One commonly used approach is to derive the number of restriction sites from the genome of a related species and then extrapolate to the focal species based on knowledge of their relative genome sizes [8]. Alternatively, guanine-cytosine (GC) composition can be determined using sequencing-independent methods such as flow cytometry [9, 10] and used to predict the frequency of a given restriction site [11]. However, both approaches have their drawbacks, and predictions based on GC content in particular do not always appear to be reliable [12, 13].

Fortunately, the low cost and relative ease of transcriptome sequencing has led to rapid growth in the numbers of transcriptome assemblies available for non-model species [14–16]. Accordingly, Herrera et al. [17] recently developed the PredRAD pipeline (available at [18]), which uses probabilistic models to predict restriction site frequencies for a given enzyme based on the nucleotidic composition of the transcriptome (or other genomic resource). These frequencies can then be multiplied by the genome size of the organism in question to obtain the expected number of restriction sites. Four different models corresponding to four different ways of assessing nucleotidic composition are available–one is based on GC content, while the other three are based on mono-, di- or trinucleotide composition, meaning the frequency of occurrence of the four bases (A, C, G and T), of the 16 possible two-nucleotide combinations (AA, AC, AG, AT etc.) and of the 64 possible tri-nucleotide combinations (AAA, AAC, AAG, AAT etc.) respectively.

Herrera et al. [17] applied PredRAD to the genome sequences of 434 different species spanning the eukaryotic tree of life. For each species, they then compared predictions based on the models described above for 18 different restriction enzymes with direct estimates of the number of cut sites in the genome based on in silico restriction digestion. For a subset of 27 species, the pipeline was also applied to transcriptome assemblies and the resulting predictions from all three models were compared with equivalent predictions derived from genome assemblies. Overall, a strong correlation was found between the transcriptomic and genomic predictions, suggesting that PredRAD is a valuable tool for designing RADseq studies. However, predictions for a given restriction enzyme varied widely depending on the model and genomic resource used. In particular, the trinucleotide model tended to perform better for many but not all of the restriction enzymes when applied to genome assemblies, but no best model was identified for transcriptome-based predictions. Consequently, the choice of the model to use for a given combination of species, genomic resource and restriction enzyme may not always be obvious.

Another potentially important source of uncertainty concerns how well predictions from both PredRAD and in silico digestion approximate the actual number of RAD loci that are obtained when a given organism is RAD sequenced. On the one hand, estimates from in silico digestion could potentially be downwardly biased if poor quality or incomplete assemblies are used. Alternatively, a RAD dataset might contain fewer loci than expected for a number of reasons, including incomplete restriction enzymatic digestion of the samples [19] or uneven sequence coverage across the genome [20], which may prevent some loci from being built. One way to investigate this would be to directly compare both PredRAD predictions and in silico digestion estimates with the number of RAD loci assembled from an empirical RADseq dataset.

In the present study, we used the Antarctic fur seal (*Arctocephalus gazella*) as a case study to investigate this topic. Antarctic fur seals have been intensively studied at Bird Island in South Georgia since the 1980’s [21] and individual-based genetic data have been amassed for over 7000 individuals since the mid 1990’s [22]. To facilitate ongoing studies of heterozygosity fitness correlations [23–25] and mate choice [26], high quality transcriptome [27] and genome assemblies [28] have recently been developed. Both are arguably more complete than is the case for many non-model organisms, the transcriptome assembly being in its third iteration and incorporating data from multiple individuals, tissue types and sequencing platforms, while the genome assembly combines Illumina data from multiple mate-pair and fosmid libraries with medium coverage PacBio sequencing (for details, see the Methods). Additionally, RADseq data were recently generated for 96 fur seal individuals digested with the most commonly used restriction enzyme, SbfI [28].

Here, we first analysed the fur seal transcriptome and genome assemblies using PredRAD to predict the number of restriction sites present in the genome for 18 different enzymes. Second, we digested the reference genome in silico with the same restriction enzymes to estimate the number of cutting sites present in the genome. For comparison, we then quantified the number of RAD loci that could be de novo assembled with seven different combinations of parameter settings and mapped to the reference genome. We hypothesised that restriction site frequencies predicted from the transcriptome and genome assemblies would be highly concordant with one another but would vary with the model used by predRAD. Our main goal was to ‘ground truth’ the various predictions by comparison to empirical estimates of the number of RAD loci obtained by assembling the RADseq data.

## Methods

### Data sources

For this study we utilized existing genomic resources and RAD data as described below. The transcriptome assembly (available at [29]) is described by Humble et al. [27]. Initially, cDNA from the skin of twelve fur seals was 454 sequenced, yielding a total of 1,443,397 reads. These were de novo assembled using Newbler into 23,025 isotigs of average length 854 bp, which in turn clustered into 18,576 isogroups [30]. To increase the representation of different tissue types, we then 454 sequenced cDNA from testis, heart, spleen, intestine, kidney and lung tissues obtained from nine adult male seals that died of natural causes [31]. This generated a further 1,046,221 reads, which when jointly assembled with the previous data resulted in a total of 23,096 contigs of mean length 971 bp. Finally, the original cDNA library was Illumina sequenced to generate 17,894,042 paired-end reads [27]. These were assembled using SOAPdenovo into 26,266 contigs of mean length 904 bp, which were then BLASTed to the 454 backbone. This allowed us to identify a further 5452 contigs that were absent from the original 454 assembly but which revealed sequence homology to the Weddell seal (*Leptonychotes weddellii*) and walrus (*Odobenus rosmarus*). These were then concatenated to the original 454 transcriptome to yield a hybrid transcriptome comprising 28,548 contigs of average length 950 bp and a combined length of 27,107,654 bp.

The reference genome assembly (available at the European Nucleotide Archive under BioProject ID PRJEB26995) is described in detail by Humble et al. [28]. Initially, high molecular weight DNA from an adult female that died of natural causes was used to construct five paired-end libraries with 180–230 bp insert sizes plus seven mate-pair libraries with 3–15Kb insert sizes and one 40 kb fosmid library [27]. These were then Illumina sequenced to generate 598 Gb of data, equivalent to ~200× depth of coverage over a 3 Gb genome. De novo assembly within Allpaths-LG resulted in 144, 410 contigs integrated within 8126 scaffolds (assembly size: 2.41 Gb; scaffold/contig N50: 3.1 Mb/27.5 kb). Subsequently, the same sample was sequenced on 64 PacBio RSII SMRT cells using P6–C4 chemistry, which yielded a total of 58 Gb (~19×) of sequencing data. PBJelly v15.8.24 and blasr were then used to align the PacBio reads to the Illumina assembly to generate a hybrid genome assembly with a total length of 2.3 Gb comprising 6169 scaffolds with an N50 of 6.2 Mb.

The RAD dataset we analysed was generated by Humble et al. [28] and comprises a total of 136,537,558 raw reads corresponding to 96 RAD sequenced fur seal individuals. These comprised adult males, adult females and pups sampled using standard methodology [32] during 1994–2002 inclusive, mainly from Bird Island, South Georgia, but also from several other islands across the species global range (the South Shetlands, Bøuvetoya, Heard Island, Isles Kerguelen and Macquarie Island). 800 ng of intact genomic DNA from each sample was digested with 20 units of SbfI prior to the ligation of P1 adapters containing unique 5-base barcodes. Uniquely barcoded samples were then pooled into six RAD libraries and sheared to ∼400 bp on a Covaris S2 sonicator. For each library, fragments in the size range ∼300–700 bp were excised from an agarose gel and the P2 adapters were then ligated. Each library was then subjected to 16–17 cycles of PCR enrichment, followed by agarose gel size selection of the ∼300–700 bp fraction. Finally the libraries were 250 bp paired-end sequenced on two Illumina HiSeq2500 lanes. Read quality was then evaluated using the software FastQC [33] and no trimming was required as the per-base phred quality score was greater than 36 at all positions. The raw data are available via the Short Read Archive (BioProject ID PRJNA473050, SRA accession SRP148937).

### Prediction of restriction enzyme cutting site numbers

The PredRAD pipeline was applied to the fur seal transcriptome and genome assemblies following the developer’s protocol [18] to predict the number of cutting sites for 18 different restriction enzymes. To compare the results obtained from the transcriptome and genome assemblies, we then carried out linear regressions separately for each model with the genome-based predictions treated as response variables and the transcriptome-based predictions treated as predictor variables. Additionally, we calculated the mean squared error (MSE), a measure that when close to zero indicates high similarity between genomic and transcriptomic predictions [17]. Finally, we used the script “restriction_site_search.sh” [18] to digest the reference genome in silico to estimate the number of restriction sites for each of the 18 enzymes. The resulting values were then used as response variables in linear regressions of the predicted values obtained from the transcriptome and genome assemblies.

### Estimation of the empirical number of RAD loci

In order to estimate the empirical number of RAD loci, the raw reads described above were de novo assembled and mapped to the reference genome. This involved a number of steps, which are summarised in Fig. 1 and described below. Briefly, our starting assumption was that two DNA fragments are generated per restriction site and that the first 250 bp of each of them is sequenced in multiple copies in each of the samples in the RAD library (Fig. 1a). As the RAD sequence data were delivered as a collection of sequence reads in fastq format with no information regarding the genomic region they come from (Fig. 1b), we then assembled the reads de novo into loci (Fig. 1c) using the Stacks pipeline [34].

Briefly, Stacks first processes samples individually to assemble raw reads into stacks, which are definable as sets of reads that originated from the same genomic location within the same sample. Subsequently, consensus sequences constructed from different individuals are used to assemble a catalogue of loci. To explore the robustness of our results to parameters used for the de novo assembly, we constructed seven different assemblies using different combinations of the key parameters -m, −M and -n, which correspond to the minimum number of reads required to build a stack, the maximum number of mismatches allowed between reads when building a stack and the maximum number of mismatches allowed between stacks consensus sequences when constructing a locus, respectively. Specifically, we used the default values of the Stacks pipeline (m = 3, M = 2 and n = 1) together with six additional sets of parameters obtained by increasing and decreasing the value of each parameter by one unit while keeping the other two parameters fixed to the default value. This approach allowed us to investigate how changing these three parameters within realistic bounds influenced the outcome of the de novo assembly process. Then, separately for each of the seven assemblies, we mapped the sequences of the loci obtained to the reference genome (Fig. 1d) using BWA [35].

Finally, we used information present in the SAM file outputted by BWA to enumerate the number of SbfI cutting sites associated with assembled RAD loci. As SAM files contain detailed information about the mapping of each specific sequence to the reference genome, they can be used to determine the genomic location of each locus in relation to known SbfI recognition sites. In this way, we could distinguish among the five different scenarios summarised in Fig. 1d. First, two independently assembled loci map to either side of a restriction site (scenario I). Second, multiple loci map to both sides of the restriction site (scenario II). This occurs because in certain genomic regions the number of polymorphisms is greater than M and / or n, resulting in homologous loci being assembled separately. Third, one or more loci map to only one side of a restriction site (scenario III). Alternatively, a pair of loci or a single locus can map to a genomic location that does not contain a cut site (scenarios IV and V) and we interpreted these as not being genuine RAD loci, even if we cannot exclude that scenario IV could sometimes represent true restriction sites containing a polymorphism. We therefore conservatively estimated the empirical number of restriction sites represented by de novo assembled RAD loci as the sum of occurrences of scenarios I–III inclusive. The processing of SAM files was conducted within R 3.4.3 [36].

## Results

### Predicted numbers of restriction sites

**Fig. 2:**
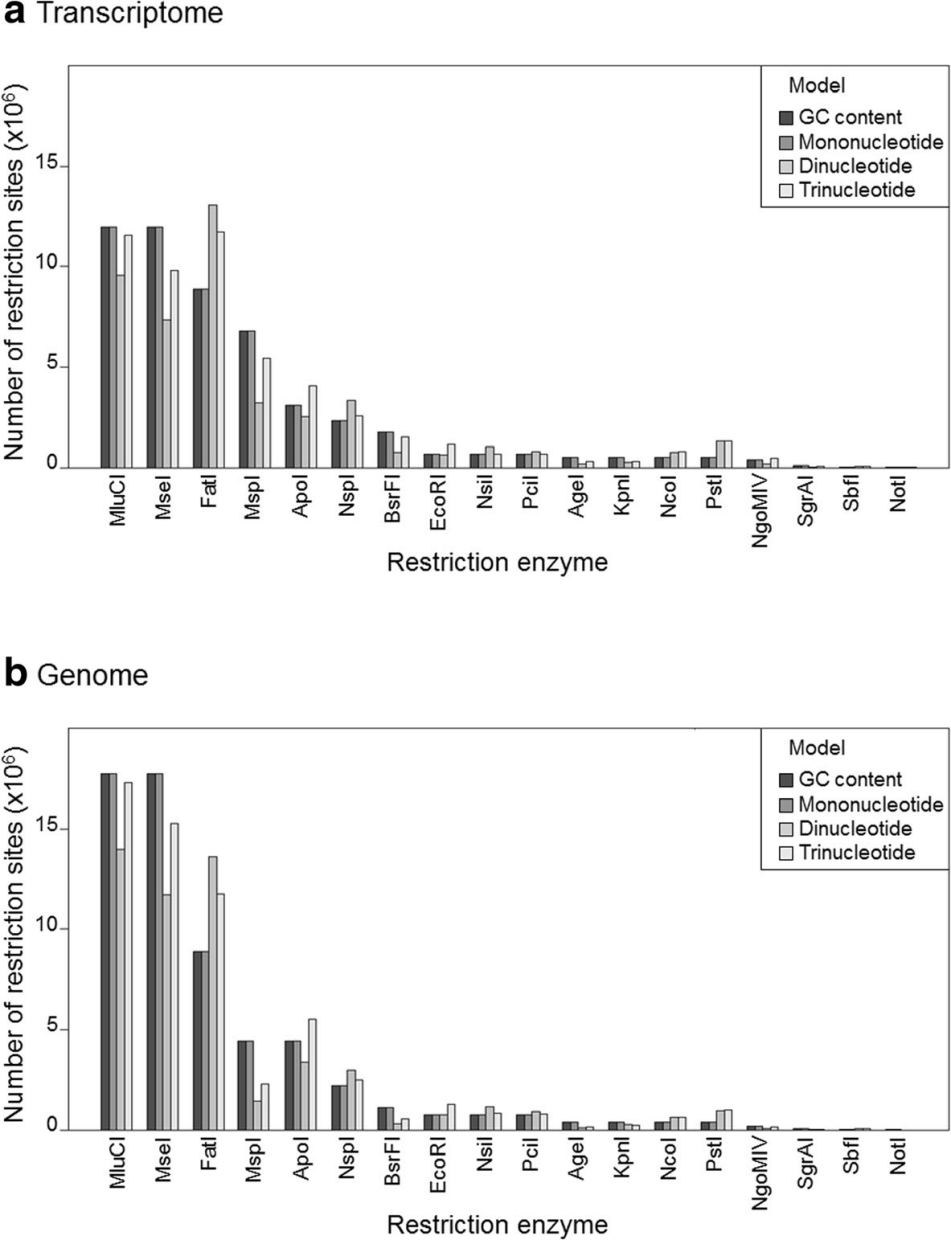
Barplot showing variability in the predicted numbers of cutting sites across 18 restriction enzymes based on (**a**) the transcriptome assembly and (**b**) the genome assembly, calculated using four different probabilistic models implemented in PredRAD

The predicted number of cutting sites in the Antarctic fur seal genome, obtained by applying the PredRAD pipeline to the transcriptome and genome assemblies, was highly variable across the 18 different restriction enzymes (Fig. 2 and Additional file 1). Predicted restriction site numbers ranged from 5136 to 13,473,997 based on the transcriptome assembly and from 762 to 17,175,211 based on the genome assembly. Predictions from the four different models were in general similar for a given restriction enzyme, with values estimated using the GC content model and the mononucleotide model being virtually identical, as also observed by Herrera et al. [17].

**Fig. 3:**
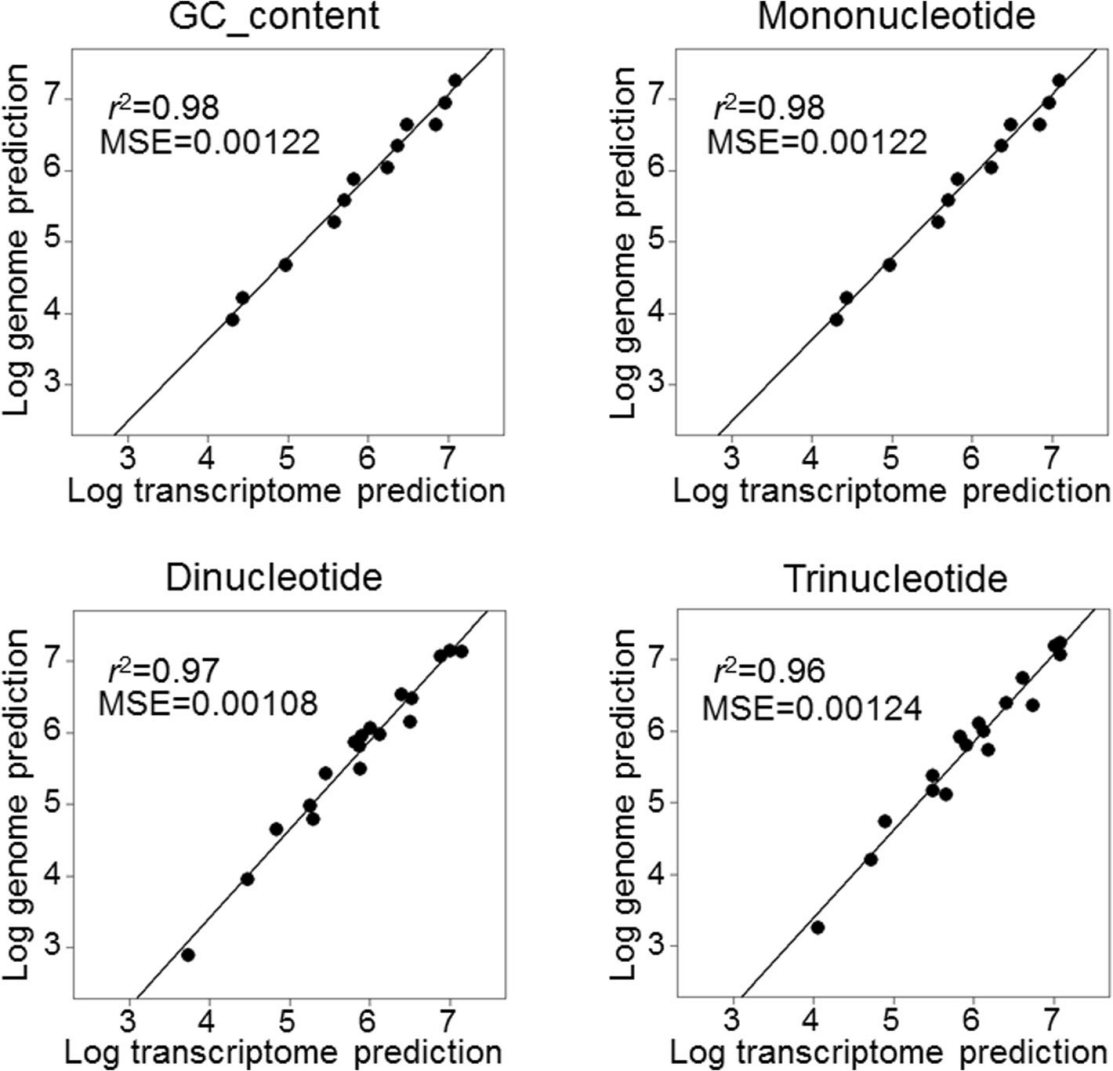
Linear regressions showing relationships between genome-based and transcriptome-based predictions for the four models (all of the models yielded *p*-values < 0.01) together with their MSE values

**Fig. 4:**
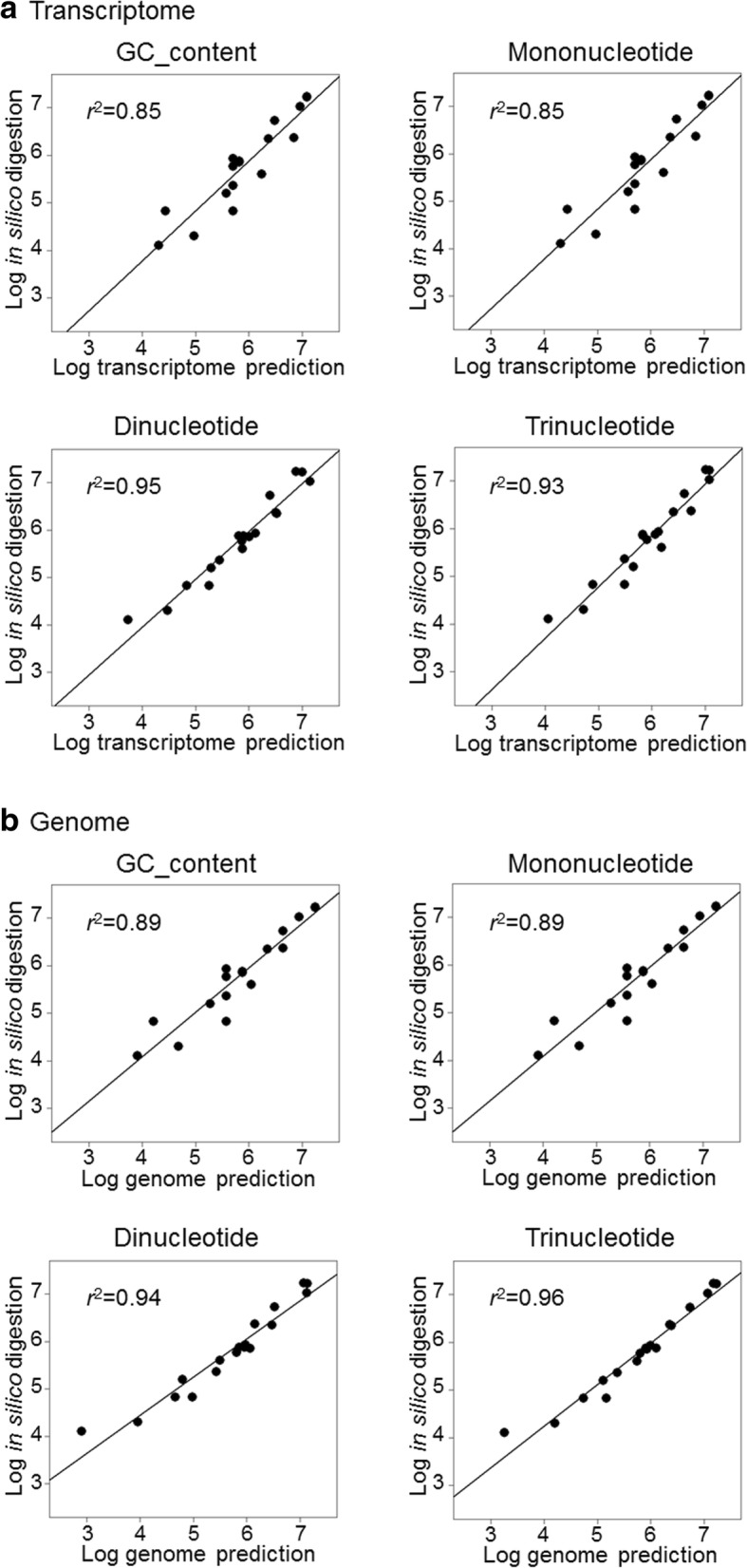
Linear regressions showing relationships between in silico observed cutting sites and (**a**) transcriptome-based predictions and (**b**) genome-based predictions for the four models (all of the models yielded *p*-values < 0.01)

Predicted numbers of restriction sites based on the transcriptome and genome assemblies were strongly correlated regardless of the specific model used, with *r*^2^ values being consistently above 0.96 after logarithmic transformation of the data to assure homoscedasticity (Fig. 3). Consistently, MSE values were all around 10^− 3^, indicating a high degree of similarity between recognition sequence frequencies predicted from the two assemblies, with the dinucleotide model showing the value closest to zero (MSE = 0.00108, Fig. 3). Restriction site numbers estimated by in silico digestion of the reference genome (Additional file 2) were also strongly correlated with PredRAD predictions from both the transcriptome and the genome assemblies, with *r*^2^ values ranging from 0.85 to 0.96 depending on the model used (Fig. 4). Specifically, the strongest correlations were obtained from the dinucleotide model for the transcriptome assembly (*r*^2^ = 0.95) and from the trinucleotide model for the genome assembly (*r*^2^ = 0.96).

### Estimated numbers of SbfI restriction sites based on in silico digestion and mapping of de novo assembled RAD loci

**Fig. 5:**
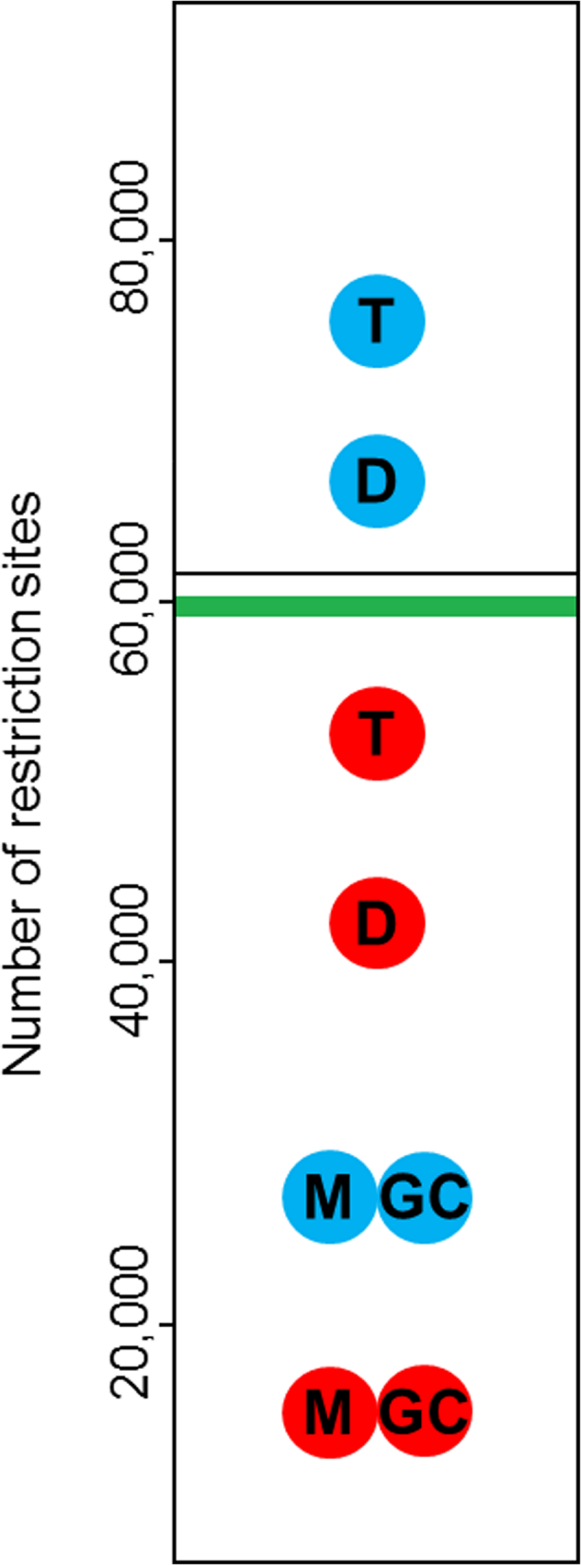
Predicted and empirical estimates of the number of SbfI restriction sites in the Antarctic fur seal genome. The continuous line corresponds to the estimate based on in silico digestion of the genome assembly. The green area shows the range in which estimates from the seven mapped de novo assemblies of the RADseq dataset are contained. Blue and red circles correspond to transcriptome-based and genome-based predictions respectively, with the codes “GC”, “M”, “D” and “T” representing predictions from the GC content, mononucleotide, dinucleotide and trinucleotide models respectively

We first estimated the empirical number of restriction sites in the fur seal genome by in silico digesting the reference genome with SbfI. This resulted in an estimated total of 61,687 restriction sites (black line in Fig. 5). For comparison, we analysed a dataset of 136,537,558 raw reads corresponding to 96 fur seals digested with SbfI to create seven different sets of de novo assembled loci based on plausible combinations of three main parameters within the Stacks pipeline. The parameter settings used together with summary information for each of the resulting assemblies are shown in Table 1. While observed heterozygosity and average depth of coverage were reasonably consistent across assemblies, ranging from 0.08 to 0.13 and from 9.46× to 11.48× respectively, considerable variability was found in the total number of assembled loci, which ranged from 276,717 to 674,500 (Table 1). To explore this further, we mapped all seven sets of loci to the reference genome. Mapping success varied from 71 to 76.63% (Additional file 3). We then quantified the number of cut sites in the genome to which the de novo assembled RAD loci mapped. RAD loci that did not map to restriction sites (scenarios IV and V in Fig. 1) were discarded. The resulting estimates of the number of SbfI restriction sites associated with RAD loci were remarkably consistent across the assemblies, averaging 59,753 and differing by at most a few hundred loci between different assemblies (Additional file 3).

**Table 1:** Summary information for the seven de novo assembled sets of RAD loci

m	M	n	Number of RAD loci	Number of RAD loci present in at least 2 individuals	Heterozygosity	Average depth of coverage
2	2	1	674,500	267,069	0.09	9.46
3	1	1	423,664	227,999	0.08	10.12
3	2	0	497,830	278,376	0.13	10.55
3	2	1	378,310	208,702	0.09	10.55
3	2	2	349,082	193,859	0.08	10.55
3	3	1	343,062	198,718	0.09	10.75
4	2	1	276,717	186,925	0.09	11.48

The Stacks parameters -m and -M define the minimum number of raw reads and the maximum number of mismatches between raw reads when creating a stack within the same individual respectively. -n corresponds to the number of mismatches allowed between stacks when processing multiple individuals to construct RAD loci. For each tested combination of parameters, we report the total number of RAD loci, the number of RAD loci present in at least two individuals, observed heterozygosity (outputted by the population module within the Stacks pipeline) and average depth of coverage

In order to explore why only a proportion of the de novo assembled RAD loci successfully mapped to the reference genome, we focused on the de novo assembly using default parameters and regressed the number of samples in which each locus was assembled on mapping probability. We found a strong positive relationship, with RAD loci that were assembled in one or only a few individuals being significantly less likely to map (Additional file 4; logistic regression, β = 0.013 ± 0.0001, *p* < 0.01). This suggests that loci assembled in larger numbers of individuals are more likely to be genuine.

To facilitate comparison of the various predictions and empirical estimates, we summarized all of the values in Fig. 5. While estimates from both in silico digestion (denoted by the black line) and de novo assembly (denoted by the green shaded area) were similar, greater variability was observed in the PredRAD predictions. Regardless of whether the transcriptome assembly (depicted by blue circles) or the reference genome (depicted by red circles) was used as an input, the predicted number of restriction sites was consistently lowest for the mononucleotide and GC models, intermediate for the dinucleotide models and highest for the trinucleotide model. Moreover, for a given model, transcriptome-based predictions were consistently higher than genome-based predictions. Overall, the two predictions that were closest to the empirical estimates of the number of restriction sites were those obtained from the dinucleotide and trinucleotide models when applied to the transcriptome and genome assemblies respectively.

## Discussion

One of the most important factors affecting the success of a RADseq project is the choice of which restriction enzyme to use, as this determines the number of RAD loci that will be obtained and consequently the depth of sequencing coverage that should result from a given sequencing effort. The PredRAD pipeline was created to facilitate this choice, allowing the estimation of restriction site frequencies from transcriptome assemblies and other genomic resources. Here we exploited a model marine mammal species for which high quality transcriptome and genome assemblies are available to compare the number of restriction sites predicted by PredRAD for 18 different enzymes with estimates based on in silico digestion of the reference genome. However, empirical RADseq datasets might not necessarily contain the expected number of loci due to factors such as incomplete enzymatic digestion, uneven depth of sequencing coverage and assembly errors. Consequently, we compared PredRAD predictions for the restriction enzyme SbfI with estimates of the number of restriction sites obtained by digesting the reference genome in silico and by assembling and mapping a RADseq dataset. Overall, we found strong concordance between transcriptomic and genomic predictions across the four models implemented in PredRAD. The resulting predictions were also highly concordant with equivalent estimates based on in silico genome digestion, indicating that PredRAD generates useful predictions for the design of RADseq projects. However, in the case of SbfI, the predicted number of restriction sites derived from the GC, mono-, di- and trinucleotide models varied considerably from under 20,000 to around 75,000. By contrast, the two empirical estimates were highly concordant with one another, at around 60,000 restriction sites. By implication, the predictions obtained from PredRAD for the dinucleotide model applied to transcriptome assembly and the trinucleotide model applied to the genome assembly appear to be closest to the most likely true value.

### PredRAD predictions

Considerable variation was observed in the predicted numbers of restriction sites across the different restriction enzymes, regardless of whether the PredRAD pipeline was applied to the transcriptome or the genome assembly. This pattern is to be expected and reflects underlying variability in the length and nucleotide composition of the recognition sequences of each restriction enzyme. In general, the most frequent cutting enzymes had shorter recognition sequences, as previously shown by Herrera et al. [17]. The same overall pattern was obtained independently of which model was used, and this pattern was also confirmed by in silico digestion.

Predictions from the transcriptome and genome assemblies were also strongly positively correlated, with *r*^2^ values ranging from 0.96 for the trinucleotide model to 0.98 for the GC content and mononucleotide models. The greatest similarity between predictions from the two assemblies was obtained for the dinucleotide model (MSE = 0.00108). This is in contrast to Herrera et al. [17], who found the best concordance with the mononucleotide model when analysing 27 different species. One possible explanation for this could be that the Antarctic fur seal transcriptome assembly is unusually complete, having been sequenced with both 454 and Illumina HiSeq technologies and assembled from multiple individuals and tissues. This would be consistent with one of the higher complexity models (the dinucleotide model) yielding the best outcome. However, the strength of correlation between transcriptomic and genomic predictions based on different models may also vary across species.

### Estimated numbers of SbfI restriction sites

Even though the predictions we obtained from PredRAD correlated strongly with equivalent in silico estimates, the empirical number of loci assembled from a RADseq dataset could potentially be different due to methodological issues such as incomplete restriction digestion or erroneous de novo assembly. We therefore decided to ‘ground truth’ PredRAD predictions for the restriction enzyme SbfI by de novo assembling RADseq data from 96 individuals and mapping the resulting loci to the reference genome to enumerate the corresponding number of restriction sites. As the de novo assembly of RADseq datasets can be sensitive to the values of three main parameters, −m, −M and -n, we constructed seven alternative assemblies, each with a different parameter combination. As expected, considerable variation was found in the number of RAD loci present in the different de novo assemblies. However, when the assembled loci were mapped to the reference genome, the number of restriction sites represented by scenarios I, II and III were highly consistent across the assemblies and very similar to the estimated number of restriction sites obtained by in silico digestion.

A probable explanation for the apparent disparity between the number of de novo assembled RAD loci and the estimated number of restriction sites in the genome could be that the former may be inflated due to assembly artefacts. Two lines of evidence support this possibility. First, the number of assembled RAD loci was highly dependent on the parameter settings used, a pattern that is intrinsic to most if not all RADseq datasets [37]. Second, we found that a large proportion of the loci that did not map to the reference genome were assembled in just one or a handful of individuals, suggesting that many of these loci are likely to be assembly artefacts. This observation is important because it suggests that a reference genome should wherever possible be used to improve the quality of a de novo assembled RADseq dataset, as recently also suggested by Shefer et al. [38]. Of course, mapping the de novo assembled loci to the reference genome places an upper limit on the number of restriction sites that can be quantified from the RAD data. However, the true number of restriction sites is unlikely to be much higher than our estimates as our reference genome shows a high degree of completeness as indicated by the very low rate of gaps (0.5%) and the fact that additional PacBio sequencing did not appreciably increase the total assembly length. Nevertheless, the number of restriction sites represented by mapped RAD loci was a little lower than our in silico digestion estimate. Hence, it would appear that not every restriction site in the genome was captured by our empirical RADseq dataset, although the vast majority were present, suggesting that it may be possible to recover most of the RAD loci present a given species’ genome.

### Comparison between PredRAD predictions and empirical estimates

Comparing predictions from PredRAD with our empirical estimates of the number of SbfI restriction sites in the fur seal genome revealed a number of patterns. First, the GC content and mononucleotide models gave by far the lowest estimates of the number of restriction sites, at around 15–25,000 sites depending on whether the transcriptome or genome assembly was used. By contrast, the dinucleotide and trinucleotide models were much closer to our empirical estimates, at around 40–75,000 restriction sites. The same pattern was found by Herrera et al. [17], who argued that the di- and trinucleotide models produce better predictions because more parameters are used (i.e. 16 two-nucleotide combinations and 64 tri-nucleotide combinations) together with longer k-mer lengths.

Second, we found that both the di- and trinucleotide models slightly underestimated the number of SbfI restriction sites when applied to the genome assembly and slightly overestimated the number of restriction sites when applied to the transcriptome assembly. This finding is consistent with trends reported for this enzyme by Herrera et al. [17]. In mammals, SbfI restriction sites are more likely to occur in conserved genomic elements than would be expected by chance [17] and these conserved elements (sensu [39]) have been identified as evidence of functional regions under purifying selection [40]. Therefore, it is plausible that the transcriptome assembly could have overestimated the number of SbfI sites due to enrichment (relative to the reference genome) for conserved functional elements.

Third, the best prediction based on the reference genome was obtained from the trinucleotide model, as observed also by Herrera et al. [17], while the best transcriptome-based prediction was obtained from the dinucleotide model. As mentioned above, this may be due to the high degree of completeness of the fur seal transcriptome assembly, which could potentially provide a relatively precise estimation of the genomic nucleotidic composition and in turn produce better estimates when a probabilistic model more complex than the GC content or mononucleotide model are applied.

## Conclusions

We compared PredRAD predictions of the number of SbfI restriction sites with empirical estimates obtained from both in silico digestion and de novo assembly and mapping of RAD loci. We found that our empirical dataset yielded inflated estimates of the number of RAD loci after de novo assembly. However, when these loci were mapped to the reference genome, the resulting number of restriction sites captured by the RAD data was consistently close to our empirical estimate from in silico digestion. This suggests that care should be taken when assembling RADseq data without a reference genome, while at least for our empirical RADseq dataset, we were able to capture loci corresponding to the vast majority of described restriction sites. However, our study was based on a single species for which high quality genome and transcriptome assemblies are available. Similar comparisons between predicted and empirical values in other species belonging to different taxonomic groups and showing differences in their genomic architecture will allow further exploration of the performance of the four distinct probabilistic models.

## Additional files

Additional file 1: Predicted numbers of cutting sites for 18 restriction enzymes based on (a) the transcriptome and (b) the genome assemblies, calculated using the four probabilistic models implemented in PredRAD. (XLSX 15 kb)

Additional file 2: In silico estimates of the number of cutting sites for 18 restriction enzymes. (XLSX 11 kb)

Additional file 3: Summary information for the alignments of the RAD locus consensus sequences obtained from the seven de novo assemblies to the reference genome. For each tested combination of parameters, we report the number and percentage of mapped RAD loci, average mapping quality, the number of RAD loci mapped to left and right sides of restriction sites separately, the number of genomic locations with RAD loci mapped to both sides of restriction sites, the number of genomic locations with RAD loci mapped to only one side of restriction sites, the number of occurrences of scenarios I, II and III, and finally the estimated number of restriction enzyme cutting sites. (XLSX 11 kb)

Additional file 4: Logistic regression of RAD locus mapping probability as a function of the number of samples in which the RAD locus was found. The red line shows the probability of mapping and the bars represent the number of RAD loci present in a certain number of samples that either mapped (upper part of the plot) or did not map (lower part of the plot) to the reference genome. (TIFF 951 kb)

## Abbreviations

GC: Guanine-cytosine
MSE: Mean squared error
RADseq: Restriction site associated DNA sequencing
SNP: Single nucleotide polymorphisms
