# Supplementary figures and images for: Experimental validation of in silico predicted RAD locus frequencies using genomic resources and short read data from a model marine mammal
Source: BMC Genomics. 2019 Jan 22;20:72. doi: 10.1186/s12864-019-5440-8 (PMC6341687; doi:10.1186/s12864-019-5440-8)

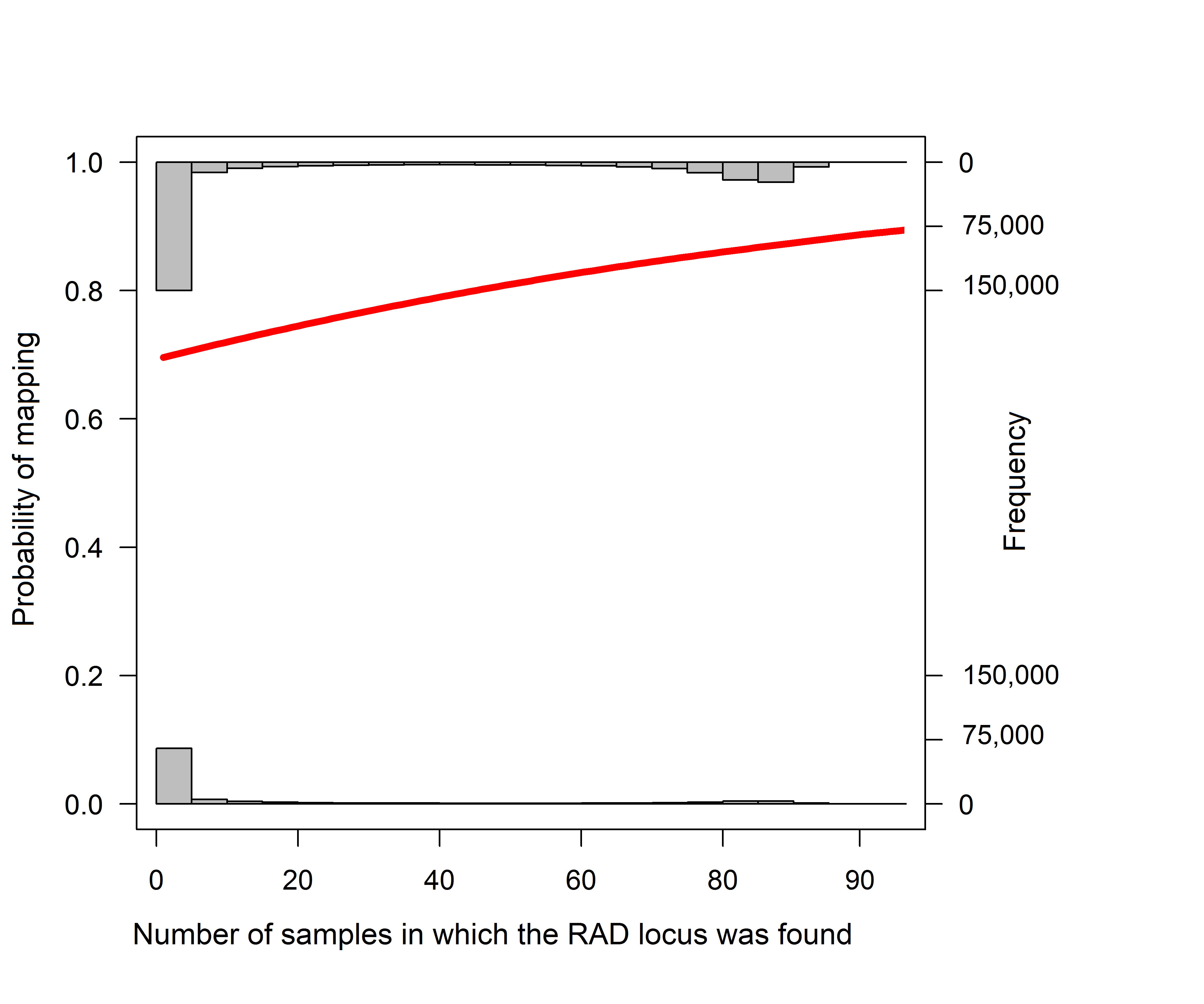

Supplement: Supplementary file 4 — Logistic regression of RAD locus mapping probability as a function of the number of samples in which the RAD locus was found. The red line shows the probability of mapping and the bars represent the number of RAD loci present in a certain number of samples that either mapped (upper part of the plot) or did not map (lower part of the plot) to the reference genome. (TIFF 951 kb) [file 12864_2019_5440_MOESM4_ESM.tiff]
